# Prediction of Helmet Use Behavior among Motorcyclists Based on the Theory of Planned Behavior

**DOI:** 10.34172/jrhs.2022.99

**Published:** 2022-12-29

**Authors:** Forouzan Rezapur-Shahkolai, Ehsan Vesali-Monfared, Majid Barati, Leili Tapak

**Affiliations:** ^1^Department of Public Health, School of Public Health, Hamadan University of Medical Sciences, Hamadan, Iran; ^2^Research Center for Health Sciences, Hamadan University of Medical Sciences, Hamadan, Iran; ^3^Social Determinants of Health Research Center, Hamadan University of Medical Sciences, Hamadan, Iran; ^4^Department of Biostatistics, School of Public Health, Hamadan University of Medical Sciences, Hamadan, Iran; ^5^Modeling of Noncommunicable Diseases Research Center, Hamadan University of Medical Sciences, Hamadan, Iran

**Keywords:** Health promotion, Injury prevention, Road traffic injury, Safe behavior, Safety promotion

## Abstract

**Background:** Road traffic injuries (RTIs) are one of the most critical factors that endanger human health. More specifically, head and neck injuries are the main causes of deaths and disabilities among motorcyclists. This study aimed to investigate the predictive factors of helmet use behavior among motorcyclists based on the theory of planned behavior (TPB).

**Study Design:** This study followed the cross-sectional design.

**Methods:** This study was conducted on randomly selected 730 motorcyclist employees in Qom, Iran, in 2021. The data collection tool was a self-administered researcher-made questionnaire, including items on demographic characteristics, history of RTIs, and constructs of TPB. Data were analyzed using descriptive summary statistics, analysis of variance, independent samples *t* test, Pearson correlation coefficient, and structural equation modeling (SEM).

**Results:** In this study, only 9.8% of the participants reported that they always used a helmet while riding a motorcycle. About 60% reported a history of a motorcycle crash, and 11.5% had a history of head injuries. The direct effect of attitude, subjective norms, and perceived behavioral control on the intention to use a helmet were statistically significant, explaining 59% of the variation in behavioral intention (intention to use a helmet) (R^2^=0.59). Moreover, perceived behavioral control and behavioral intention had significant effects on helmet use behavior (R^2^=0.26).

**Conclusion:** The prevalence of helmet use among the studied population was very low. Moreover, TPB was useful in identifying the determinants of behavior and especially behavioral intention of helmet use among motorcyclists.

## Background

 Road Traffic Injuries (RTIs) are among the most important public health threats that have endangered people’s health.^1^ According to the World Health Organization (WHO) reports in 2018, more than 3500 daily deaths and more than 1.3 million yearly deaths occurred due to RTIs. Moreover, it has been reported that 20 to 50 million people suffer from non-fatal injuries globally. In low- and high-income countries, it has been reported that RTI causes about 27.5 and 8.3 deaths per 100 000 population, respectively.^2^

 Based on the WHO report in 2015, RTIs have been known as the ninth cause of mortality and disease burden globally, and it has been estimated that by continuing the current trend until 2030, it will jump to the fifth rank.^3^ In developing countries, 80% of mortalities and 90% of morbidities are related to RTIs.^4^ Noticeably, head and neck injuries/trauma are the main causes of mortality, intense injuries, and disability among motorcyclists.^5^

 Based on the WHO report in 2018, the distribution of mortality due to RTIs in Iran reveals that the motorized 2-3 wheelers death rate is 24.1%.^2^ Studies conducted in Iran in 2019 have shown that the prevalence of RTIs is about 20 times more than the global average.^6^ Other studies revealed that over 25% of mortalities due to RTIs are related to motorcycle crashes.^7,8^ The report given by the WHO emphasizes that wearing a helmet could decrease mortality risk by 42% and the risk of intense injuries by about 70%.^3^ In Iran, since 2001, motorcyclists must wear a helmet based on the regulation of traffic, and the Traffic Police are obliged to enforce the legislation.^7^

**Figure 1:**
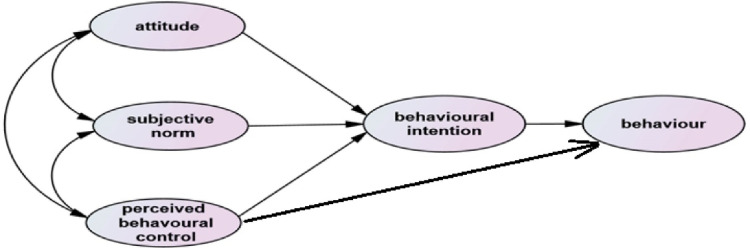
Schematic representation of the theory of planned behavior

 Despite the importance of helmet use, the rate of its use is different in Iran.^9^ Therefore, there is an urgent need to study the rate of helmet use and to identify the predictors of helmet use behavior among Iranian motorcyclists. On the other hand, theories and behavioral models can be helpful to identify predictive factors. One of the most prevalent and suitable models is the theory of planned behavior or TPB (Figure 1). Many studies have shown that this theory could be applied to the prediction of traffic intention and behavior.^10-12^

 Based on TPB, intention predicts behavior and is itself influenced by attitude, subjective norms, and perceived behavioral control. Attitudes indicate an individual’s positive/negative evaluation of a specific behavior. Subjective norms are related to perceived social pressure that might lead to performing or not performing a specific behavior, and finally, perceived behavioral control is the perception of each individual about the difficulty of doing a specific behavior.^13^

 Considering the importance of helmet use in preventing severe head injuries among motorcyclists and the identification of factors predicting helmet use behavior, this study aimed to predict the factors affecting helmet use behavior among motorcyclists. It can help to design more appropriate and evidence-based intervention programs.

## Materials and Methods

###  Study design and setting 

 This cross-sectional study was conducted in 2021 (July 1st-August 30th) among the employees of government institutions and offices in Qom, Iran. The city of Qom with an area of 11 237 square kilometers is located 120 km to the southwest of Tehran (the capital of Iran).^14^ Considering the climate and weather of Qom, the metropolitan population, and the cheapness of motorcycles, compared to cars, as well as the location of government institutions and offices placed on routes that are part of high-traffic urban areas, traveling by private car during the day has been restricted by the police. Since it is easier and faster for employees to reach their workplace by motorcycle, their use is common among employees in this city. Therefore, the present study was carried out among this group.

###  Sampling

 The method of sampling was stratified random sampling, and the research population included employees in governmental institutions and offices of Qom, who commute to work by motorcycle. All participants were males since females do not use a motorcycle for commuting in the context of Iran. For this purpose, all governmental offices/institutions of Qom were considered as the strata (the employees who work in an office are homogeneous in terms of behavior), and the employees from each office were selected randomly proportionate to the number of employees of that office using motorcycles for commuting.

 The confidence interval was considered 95% (1-α = 0.95), and the prevalence of helmet use behavior was estimated at 21.5%, based on previous studies.^15^ Additionally, the relative estimation error was considered 9.8% of the prevalence, so the total sample size was calculated as 730, including a 10% non-response rate. Inclusion criteria were (1) being an employee in a governmental institution/office, (2) having a motorcycle driver’s license, (3) using and riding a motorcycle to commute to work for at least six months, and (4) having informed consent to participate in the study. On the other hand, participants who were on vacation/leave at the time of data collection were excluded from the study.

###  Data collection instrument and strategy

 Data were collected using a researcher-made questionnaire that was prepared based on previous studies, and its validity and reliability were also checked.^11,16-18^ Completion of the questionnaire was also done in a self-administered manner. The first part of the questionnaire included questions about demographic characteristics, such as age, marital status, education level, history of being fined by the police, history of motorcycle crashes, history of injury and head trauma, as well as hospitalization and/or having surgery at the hospital. The second part, was designed based on the constructs of the TPB, including questions on attitude, subjective norms, perceived behavioral control, behavioral intention, and helmet use behavior among employees who were riding motorcycles. A 5-point Likert scale was used, ranging from 5 (completely agree) to 1 (completely disagree) for the constructs of attitude, subjective norms, perceived behavioral control, as well as behavioral intention, and also from 5 (always) to 1 (never) for the construct of behavior (helmet use).

 Attitude towards helmet use behavior was evaluated and measured based on six items (e.g., “I think it is very important to wear a helmet when riding a motorcycle”). The construct of subjective norms was measured based on seven items (e.g., “my family members encourage me to wear a helmet when riding a motorcycle”). The perceived behavioral control construct was measured based on eight items (e.g., “I can wear a helmet while riding a motorcycle even if it makes me feel hot or sweaty”). The behavioral intention construct was measured based on four items (e.g., “I intend to use a helmet when riding a motorcycle on the main streets and highways in the city”). The behavior (helmet use) construct was also evaluated and measured based on four items (e.g., “How often do you use a helmet when riding a motorcycle on intercity (outside the city) roads?”).

 To assess qualitative content validity, a panel of 20 experts in the fields of Health Education and Health Promotion were asked to consider compliance with grammar, the use of appropriate wording, the importance of items, the placement of items in their proper place, and the completion time of the designed tool. Furthermore, the experts were asked to assess the qualitative face validity of the instruments by commenting on the difficulty, appropriateness, and clarity of the items.

 The quantitative content validity of the questionnaire was assessed using the content validity ratio (CVR) and content validity index (CVI) to ensure that the most important and correct (the most necessary) items were selected. The questionnaire was evaluated by Health Education experts. According to the number of experts in this study and based on Lawshe’s CVR table, the items with a CVR of greater than 0.42 were reported in the questionnaire. In evaluating the CVI, the experts recorded their comments about four criteria regarding each item, including relevance, necessity, simplicity, and clarity, based on a 4-Point Likert Scale. According to the results, the CVI of all items was greater than 0.79.

 The reliability of the questionnaire was assessed using Cronbach’s alpha and intraclass correlation coefficient (ICC). For all constructs, Cronbach’s alpha was greater than 0.7 (ranging between 0.85-0.93), and the ICC was over 0.85. The details of Cronbach’s alpha value of the structures are as follows:

 (Behavior: α = 0.92, behavioral intention: α = 0.87, perceived behavior control: α = 0.93, subjective norms: α = 0.85, and attitude: α = 0.86).

###  Data analysis

 After collecting the data, they were analyzed using the SPSS software (version 23). Absolute and relative frequency were used to describe the demographic characteristics of the participants. Mean and standard deviation were used to summarize the constructs of the TPB model. Analysis of variance and independent samples t-test were used to compare the average helmet use behavior across the levels of demographic characteristics. The Pearson correlation coefficient test was used to determine the correlation between the constructs. The calculation of the mean percentage of the achievable score was based on the formula of (Mean−Minimum)÷(Maximum−Minimum) × 100.Confirmatory factor analysis was used to evaluate the measurement model. Structural equation modeling (SEM) was used to determine the predictability of the model structures and to determine the goodness-of-fit of the model using the Amos software (version 24).

###  Ethical considerations

 The present study was approved by the Ethics Committee of Hamadan University of Medical Sciences (approval code: IR.UMSHA.REC.1399.826). The participants were informed about the study, and their voluntary participation in the study and their oral informed consent were obtained. The questionnaires were anonymous, and the data were confidential.

## Results

 In this study, 685 (out of 730) employees participated (the non-response rate was 6.2%). On the other hand, those who were busy, did not have consent, and were on vacation/leave at the time of data collection did not participate. The results of the study showed that 9.8% of the participants reported that they always used a helmet while riding a motorcycle.

 About 60% of the participants reported a history of a motorcycle crash, and 11.5% had a history of head injury. Moreover, 44.1% reported being fined by the police for not wearing a helmet. A total of 331 participants (48.3%) were between 31 to 40 years (mean ± SD = 34.49 ± 7.09). A total of 597 participants (87.2%) were married, and 296 (43.2%) had a bachelor’s degree. Moreover, the results of the present study showed a statistically significant association between age (*P* < 0.013) and helmet use behavior. In addition, the relationships between helmet use behavior and the variables of helmet ownership (*P* < 0.001), history of being fined by the police for not using a helmet at the time of motorcycle riding (*P* < 0.015), and having a helmet at the time of the crash (*P* < 0.001) were statistically significant (Table 1).

**Table 1:** Frequency distribution of demographic characteristics and mean (standard deviation) of helmet use behavior score across groups (n = 685)

**Variables**	**Frequency**		**Helmet use Behavior**		***P*** **value**^a^
	**Number**	**Percent**	**Mean**	**SD**	
Age (y)					0.013
20-30	82	12.0	11.41	5.21	
31-40	331	48.3	11.05	4.86	
41-50	240	35.0	12.22	5.14	
≥ 51	32	4.7	13.18	5.53	
Marital status					0.099
Single	88	12.8	10.77	5.28	
Married	597	87.2	11.72	5.02	
Education status					0.708
Diploma	152	22.2	11.38	4.95	
Associate	130	19.0	11.75	5.39	
Bachlor	296	43.2	11.48	5.03	
Master and doctorate	107	15.6	12.05	4.93	
History of crash					0.071
Yes	413	60.3	11.32	5.07	
No	272	39.7	12.03	5.02	
Having a helmet at the time of the crash					0.001
Yes	103	15.0	15.30	4.15	
No	582	85.0	9.96	4.66	
History of a head injury					0.510
Yes	79	11.5	10.94	5.23	
No	606	88.5	11.36	5.05	
Treatment status after injury					0.296
Hospitalization and surgery	48	7.0	12.50	4.85	
Hospitalization without surgery	36	5.2	11.88	5.33	
Outpatient treatment	148	21.6	11.22	4.78	
None	453	66.2	11.01	5.34	
History of being fined by the police					0.015
Yes	303	44.1	11.07	4.69	
No	382	55.9	12.32	5.31	
Helmet ownership					0.001
Yes	287	41.9	13.63	4.50	
No	398	58.1	10.14	4.95	
Financial affordability to buy a helmet					
Yes	153	23.0	11.64	4.99	0.219
No	534	77.0	11.57	5.09	

^a^One-way analysis of variance (ANOVA) or independent samples *t* test.

 The results of Table 2 indicate the mean and standard deviation of helmet use behavior on different occasions. As this table shows,the mean score of helmet use behavior is the highest (3.27 ± 1.51) among motorcyclists while riding a motorcycle on intercity (out of town) roads, compared to other occasions.

**Table 2:** Mean and standard deviation of helmet use behaviors on different occasions among the motorcyclists (n = 685)

**Helmet Use Behavior**	**Score range**	**Mean**	**Standard deviation**
How often do you wear a helmet when you ride a motorcycle in the alleys of the inner city?	1-5	2.54	1.31
How often do you wear a helmet when you ride a motorcycle on the main streets and highways in the city?	1-5	2.98	1.44
How often do you wear a helmet when you ride a motorcycle on intercity (out-of-town) roads?	1-5	3.27	1.51
In general, how often do you wear a helmet while riding a motorcycle?	1-5	2.79	1.32
Total helmet use behavior	4-20	11.60	5.06

 The mean ± SD of the constructs of the TPB are shown in Table 3. According to the results, among the constructs of attitude, subjective norms, and perceived behavioral control, the attitude construct received the highest mean score (27.19 ± 3.5) accounting for 88.2% of the maximum achievable score. Moreover, the results indicate the mean score of helmet use intention was 78.6% of the maximum achievable score, while the mean score of helmet use behavior was 47.4% of the maximum achievable score. The results of the study revealed that the mean score of helmet use behavior is the lowest among motorcyclists while riding in inner-city streets and alleys, compared to other occasions.

**Table 3:** Mean, standard deviation, and score range of the theory of planned behavior constructs (n = 685)

**Construct**	**Mean**	**SD**	**Score range**	**Mean percentage of** **achievable score**^a^
Attitude	27.19	3.50	6-30	88.2
Subjective norms	26.32	6.30	7-35	69.0
Perceived behavioral control	29.93	7.80	8-40	68.5
Behavioral intention	16.59	3.30	4-20	78.6
Behavior	11.60	5.06	4-20	47.4

^a^Calculation: (Mean−Minimum)÷(Maximum−Minimum) × 100

Table 4 illustrates the results of Pearson correlation analysis between TPB constructs. As can be seen, all constructs of the TPB were correlated with the intention to helmet use scores and the behavior of helmet use. Among the three constructs (attitude, subjective norms, and perceived behavioral control), perceived behavioral control showed the highest correlation with the intention of helmet use (r = 0.668). Moreover, the intention of helmet use scores and perceived behavioral control were positively and significantly correlated with helmet use behavior scores.

**Table 4:** Pearson correlation coefficients between the constructs of the theory of planned behavior (n = 685)

**Construct**	**Behavior**	**Attitude**	**Subjective norms**	**Perceived behavioral control**	**Behavioral intention**
Behavior	1.000				
Attitude	0.326	1.000			
Subjective norms	0.419	0.461	1.000		
Perceived behavioral control	0.461	0.635	0.502	1.000	
Behavioral intention	0.409	0.578	0.489	0.668	1.000

 Before fitting the structural model, the measurement model was evaluated using confirmatory factor analysis. Several goodness-of-fit criteria were used to assess the model. All criteria were within acceptable limits. The ratio between χ^2^/df was obtained at 2.89, which was less than 5 (the acceptable region is < 5). The goodness-of-fit index (GFI = 0.902) and the adjusted goodness-of-fit index (AGFI = 0.878) were greater than the acceptable threshold of 0.8. The comparative fit index was also greater than the threshold value of 0.8 (CFI = 0.95). The root of the mean squares of the approximation error was less than 0.08 (RMSEA = 0.053). Therefore, according to the obtained criteria, the measurement model was favorable and was thus confirmed (CFI = 0.953, TLI = 0.945, NFI = 0.930, AGFI = 0.878, CMIN = 1018.380, df = 352, χ2/df = 2.89 GFI = 0.902, and RMSEA = .053). The combination of these indicators confirms that the measurement model fits the data and can effectively reproduce the covariance matrix.

**Figure 2:**
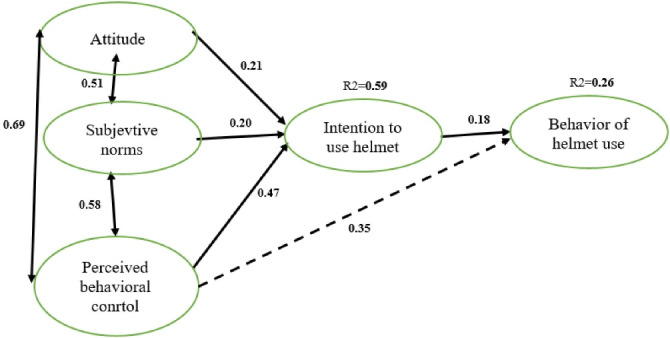
Standardized regression coefficients of the structural model of the behavior of helmet use based on the theory of planned behavior

 The results of the SEM of the TPB are given in Table 5 and Figure 2. As illustrated, the effect of attitude, subjective norms, and perceived behavioral control constructs on the behavioral intention construct was statistically significant (*P* < 0.001). In addition, the effect of behavioral intention and perceived behavioral control on helmet use behavior were statistically significant (*P* < 0.001). The results of the SEM analysis (Figure 2) revealed the direct effect of attitude construct (β = 0.21), subjective norms (β = 0.20), and perceived behavioral control (β = 0.47) on behavioral intention, where the perceived behavioral control construct had a greater contribution in predicting behavioral intention. Moreover, the perceived behavioral control predicts helmet use behavior directly (β = 0.35). According to the results shown in Figure 2 for the standardized regression coefficients, the three constructs of attitude, subjective norms, and perceived behavioral control predicted 59.0% of the intention to use a helmet (R^2^ = 0.59), where the effect of the perceived behavioral control construct was the greatest among others. Finally, perceived behavioral control, along with behavioral intention, predicted 26.0% of the variance in helmet use behavior (R^2^ = 0.26).

**Table 5:** Regression coefficients obtained using structural equation modeling for the main constructs of the theory of planned behavior (n = 685)

**Construct**	**Relationship**	**Construct**	**Estimate**	**SE**	**Test statistics**	***P*** ** value**
Attitude	→	Intention	0.350	0.076	4.600	0.001
Perceived behavioral control	→	Intention	0.548	0.059	9.334	0.001
Subjective norms	→	Intention	0.253	0.053	4.793	0.001
Perceived behavioral control	→	Behavior	0.532	0.086	6.174	0.001
Intention	→	Behavior	0.224	0.073	3.060	0.002

## Discussion

 Regarding the aim of this study to determine the predictive factors of helmet use behavior among motorcyclists based on the TPB constructs, the findings revealed the direct positive effects of attitude, subjective norms, and perceived behavioral control constructs on the intention of helmet use. In addition, 9.8% of the participants reported that they always used a helmet while riding a motorcycle. More than half of the respondents reported a history of a motorcycle crash, and about 11.5% reported a head injury history. The results of analyzing TPB constructs also showed that despite having a high level of helmet use behavior intention, the participants attained a moderate level of motorcycle helmet use behavior. Totally, TPB constructs predicted 59.0% of helmet use intention. Furthermore, perceived behavioral control and behavioral intention constructs predicted 26.0% of helmet use behavior.

 Based on TPB, the immediate cause of intended behavior is an individual’s intention to be involved in that behavior. The behavioral intention is in turn determined by individuals’ attitudes toward that behavior. For example, “using a helmet is good”.^17^ In addition to the perceived behavioral control, attitude by itself is a direct important predictor of behavioral intention.^16,19^

 In this study, we observed a statistically positive significant relationship between attitude and behavioral intention. Considering the frequency and the mean of demographic characteristics, about half of the participants had an academic education, the majority were aged 31-40 years and married, and more than half of them had a history of motorcycle crashes. It seems that demographic characteristics had an impact on the participants’ attitudes and behavioral intentions, and they considered not wearing a helmet dangerous and harmful. Consistent with our findings, Lajunen and Räsänen,^17^ Brijs et al,^16^ and Özkan et al^11^ evaluated the benefits of the TPB in motorcyclists in 2014 and confirmed the above relationships. Malekpour et al^10^ and Beck et al^20^ found a similar result between intention and attitude toward seat belt use. Malekpour et al^10^ and Torquato et al^21^ Evaluated intention and the rate of seat belt use among 18-51-year-old students in Brazil. They have shown that intention had a significant correlation with all TPB constructs, with the strongest correlation related to attitude.^21^

 Lajunen and Räsänen^17^ compared health beliefs and the TPB models regarding helmet use in the cyclists of Finland. They found that the attitude construct, along with the subjective norms, had a stronger positive significant relationship with behavioral intention. Aiming at evaluating the effect of behavioral sciences on helmet use behavioral intention among Malaysians, Ambak et al^22^ emphasized the important role of attitudes in the development of the intention to use a helmet. Given the findings of the present study, along with the results of other studies regarding the positive attitudes toward helmet use, it seems that the intention to use a helmet could be enhanced by designing targeted methods for improving individuals’ attitudes, such as encouraging cyclists to use helmets by traffic police. Moreover, as there was observed a statistically significant relationship between having a helmet and helmet use behavior, it is ideal to use financial support alongside educational activities to enhance the effect of educational interventions and improve attitudes towards helmet use and the intention to use a helmet.

 Based on TPB, behavioral intention is determined by subjective norms, including individuals’ beliefs concerning whether people who are important think if he/she should do a behavior.^17^ Our findings revealed a significant association between subjective norms and the intention to use a helmet. It seems being in an environment and exposure to susceptible and impressive situations, such as a job environment and the probability of being judged by coworkers and authorities may be effective in behavioral intentions as an abstract and subjective variable. This brings to mind that individuals’ coworkers and friends, as well as people who are important, in turn, affect the intention to use a helmet. Therefore, it is predictable that coworkers influence a person’s behavioral intentions. Aligned with the present study’s results, Quine et al^23^ found that subjective norms are a sound predictor of behavioral intentions.

 Although another study by Courneya et al^24^ showed that the direct effects of attitude and perceived behavioral control are well documented, the results for subjective norms were less consistent, and compared to attitude and perceived behavioral control, subjective norms construct was not an important predictor of behavioral intentions. Aiming at evaluating the effects of behavioral sciences on behavioral interventions for seat belt use, Ambak et al^22^ emphasized the vital role of subjective norms in the development of intention to use a seat belt.

 Kumphong et al^25^ compared the theory of reasoned action (TRA) and TPB in evaluating the effect of subjective norms on helmet use behavior. They found that subjective norms construct affects the intention to use a helmet. Nevertheless, other studies have revealed that subjective norms are among the most influential factors that explain 25.0% and 38.0% of helmet use variance based on TRA and TPB, respectively.^17^ Moreover, the findings of this study revealed that the point of view of friends, family, and those who expect an individual to use a helmet, affects the intention and behavior of helmet use. In our study, subjective norms could predict the intention to use a helmet directly and could predict the behavior of helmet use directly and indirectly through perceived behavioral control and attitude. Given the critical effects of peers and people who are important to an individual on improving helmet use behavior, it is expected that influential programs on these important groups could affect individuals’ behavioral intentions.

 The third predictor of behavioral intention is perceived behavioral control, which refers to an individual’s perception of the extent of difficulty doing a behavior. Perceived behavioral control has both direct and moderating effects (through behavioral intention) on behavior in TPB.^17^ The findings of this study revealed a significant relationship between perceived behavioral control construct and the intention to use a helmet. Among others, the perceived behavioral control construct was the strongest predictor of helmet use intention indicating that the participants believed in personal control and their ability to use a helmet. This was consistent with the findings of the study conducted by Brijs et al^16^ aiming to evaluate the psychological determinants of helmet use behavior in Cambodia. The findings of the study of Lajunen et al^17^ conducted to compare the health belief and TPB in determining helmet use behavior among the Finnish also revealed that perceived behavioral control was the most important predictor of behavioral intention.

 Contrary to our findings, the findings of a study conducted by Şimşekoğlu and Lajunen,^26^ which aimed to compare TPB and HBM to identify psycho-social factors in using a seat belt, revealed that perceived behavioral control is not a predictor of intention/behavior of using a seat belt. Aiming at developing a TPB to explain pedestrians’ violation of crossing behavior, Zhou et al^27^ conducted a study in China and concluded that perceived behavioral control was not an important predictor of pedestrians’ violation of crossing behavior. It is rational to expect that individuals are stimulated to do healthy behaviors (e.g. helmet use) and even show the behavior in dealing with challenges when they feel they have control over it.

 Given the whole structure of TPB, attitude, subjective norms, and perceived behavioral control constructs were positively and significantly associated with behavioral intention in this study. Therefore, we expect that behavioral intention could be well predicted by the constructs. Aligned with our findings, Ambak et al^22^ conducted a study aiming to apply behavioral science theory or model in predicting the intention to use helmets correctly and determining an important predictor that promotes the behavioral intention to use a helmet in Malaysia. The findings revealed that all TPB constructs had a positive and significant relationship with helmet use intention and behavior.

 Consistent with the present findings, Torquato et al^21^ conducted a study to evaluate the intention and rate of seat belt use among students aged 18 to 51 in Brazil. The findings showed that the intention to use seat belts was associated with all TPB constructs. Furthermore, Lajunen and Räsänen^17^ conducted a study to compare health beliefs and TPB regarding helmet use in cyclists in Finland. They concluded that all TPB constructs had a significant relationship with the intention to use a bicycle helmet.

 In the present study, the behavioral intention and the perceived behavioral control were directly related to helmet use behavior. Being influenced by the attitude, subjective norms, and perceived behavioral control constructs, the intention to use a helmet in other motorcyclists and coworkers may explain helmet use behavior in society. Moreover, the findings indicated that perceived behavioral control could affect the intention and behavior of helmet use in others as well. Consistent with our findings, Torquato et al^21^ concluded that intention could be substantially related to seat belt use behavior. Another study regarding the use of seat belts among students carried out in Tabriz, Iran, also revealed that the intention to use a seat belt and perceived behavioral control directly affect seat belt use behavior.^10^

 In a study conducted by Siviroj et al^28^ in Singapore, it was found that about half of motorcyclists (44.8%) had intended to use a helmet. Acheampong et al conducted a study to investigate the effect of the physical environment and personal characteristics on the intention to use a bike to go to work in Ghana and showed that among all TPB’s constructs, perceived behavioral control was the strongest predictor and had a positive direct effect on the bicyclists’ desire in using bicycle.^29^ It seems that alongside the necessity of education, providing information, and culturalization, it is essential to intensify surveillance of traffic police on motorcyclists’ behavior in using helmet according to the safety management, transportation, and driving regulations. It is necessary to put road controls on motorcyclists who do not wear a helmet and make coordination with other governmental organizations regarding the enforcement of using a helmet for motorcyclist employees as a commitment to the rules and regulations.

 Intention is a substantial predictor of behavior. Our results showed that TPB constructs could explain 59.0% and 26.0% of intention and behavior, respectively. In a study, Brijs et al^16^ revealed that TPB’s constructs could predict about 64.0% of intention and 75.0% of helmet use behavior. Tavafian et al^30^ found that TPB constructs could considerably predict the intention to use a seat belt. Aligned with our findings, Mehri et al^18^ in Yazd, Iran, showed that 59.0% of intention and 45.0% of helmet use behavior could be explained by TPB constructs. In a study conducted by Peden et al,^1^ it was found that only 46.0% of behavior was related to all constructs of TPB. The difference in the predictability of intention and behavior by TPB in the present study may indicate that the intention to use a helmet is necessary to perform the behavior, but it is not sufficient. Meanwhile, removing the potential barriers, such as the ability to buy a suitable helmet, is essential to convert their intention into behavior.

 We used a self-reporting method for data collection. In addition, the present study was carried out cross-sectionally. Since the study was conducted based on a correlational design, it was only possible to identify predictors of intention and behavior, and we could not identify the cause-effect relationships since we used correlation analysis. The study population and the studied sample were employees riding a motorcycle, so we could not generalize our findings to all societies although the sample size was large enough.

## Conclusion

 This study showed that the rate of helmet use in the studied population was very low, although helmet use is compulsory for motorcyclists. Therefore, TPB is an appropriate theory for identifying determinants of behavior and especially, the intention to use a helmet. In addition, the results of the present study could be used in designing effective interventions to promote helmet use behavior and reduce related injuries. The mentioned interventions could be effective through changing motorcyclists’ attitudes towards helmet use, modifying social norms, and increasing perceived behavioral control alongside the need to promote the surveillance of law enforcement on wearing helmets and facilitate access to helmets to establish helmet use behavior among individuals with the intention to use a helmet.

**Highlights**

- The rate of helmet use was very low among the studied population although it is compulsory for motorcyclists in Iran.
- About 60% of the participants reported a history of a motorcycle crash, and 11.5% had a history of head injury.
- There were significant relationships between helmet use behavior and variables of age, helmet ownership, being fined for not using a helmet, and having a helmet at the time of the crash.
- The TPB is useful to identify the determinants of behavior and especially, the behavioral intention of helmet use among motorcyclists.

## Acknowledgments

 The present study was approved and supported by Hamadan University of Medical Sciences. The authors would like to appreciate the Research and Technology Deputy of the Hamadan University of Medical Sciences for their approval and financial support. We also express our gratitude to all motorcyclists who participated in this study.

## Conflict of interest

 The authors declare no competing financial interests or personal relationships that could have appeared to influence the work reported in this paper.

## Funding

 This study was financially supported by the Research and Technology Deputy of the Hamadan University of Medical Sciences [Grant No. 9911077729].
